# Cryoloading: introducing large molecules into live synaptosomes

**DOI:** 10.3389/fncel.2014.00004

**Published:** 2014-01-23

**Authors:** Arup R. Nath, Robert H. C. Chen, Elise F. Stanley

**Affiliations:** Laboratory of Synaptic Transmission, Toronto Western Research InstituteToronto, ON, Canada

**Keywords:** presynaptic, synaptosome, peptide loading, intracellular, synaptic vesicle, recycling, styryl dye

## Abstract

Neurons communicate with their target cells primarily by the release of chemical transmitters from presynaptic nerve terminals. The study of CNS presynaptic nerve terminals, isolated as synaptosomes (SSMs) has, however, been hampered by the typical small size of these structures that precludes the introduction of non-membrane permeable test substances such as peptides and drugs. We have developed a method to introduce large alien compounds of at least 150 kDa into functional synaptosomes. Purified synaptosomes are frozen in cryo-preserving buffer containing the alien compound. Upon defrosting, many of the SSMs contain the alien compound presumably admitted by bulk buffer-transfer through the surface membranes that crack and reseal during the freeze/thaw cycle. ~80% of the cryoloaded synaptosomes were functional and recycled synaptic vesicles (SVs), as assessed by a standard styryl dye uptake assay. Access of the cryoloaded compound into the cytoplasm and biological activity were confirmed by block of depolarization-induced SV recycling with membrane-impermeant BAPTA (a rapid Ca^2+^-scavenger), or botulinum A light chain (which cleaves the soluble NSF attachment protein receptor (SNARE) protein SNAP25). A major advantage of the method is that loaded frozen synaptosomes can be stored virtually indefinitely for later experimentation. We also demonstrate that individual synaptosome types can be identified by immunostaining of receptors associated with its scab of attached postsynaptic membrane. Thus, cryoloading and scab-staining permits the examination of SV recycling in identified individual CNS presynaptic nerve terminals.

## Introduction

At a typical synapse in the central nervous system (CNS) a small, ~2 μm, presynaptic bouton contacts a spine or cell body of a target neuron. Classical transmitters are released by the calcium-gated fusion of synaptic vesicles (SVs) with the surface membrane. The SVs are then recovered, recycled and refilled for reuse. However, the analysis of SV recycling and fusion has been limited by the inability to reliably introduce compounds into the cytoplasm to probe molecular events. Our objective was to develop a method to load CNS presynaptic terminals with test compounds while retaining their ability to exhibit calcium-gated SV recycling.

CNS terminals can be isolated by sub-cellular fractionation as ‘synaptosomes’ (SSMs), comprising the nerve terminal together with a small scab of postsynaptic membrane, and SV recycling can be monitored by depolarization-induced uptake of green or red styryl dyes, Fei Mao (FM)1-43 or FM4-64, respectively (Bouvier et al., 1996). We introduced compounds into individual identified SSMs by “cryoloading”, an idea conceived by combining two published SSM methods: freeze-thaw, to admit the alien compounds (Nichols et al., 1989; Tamura et al., 2001) with cryopreservation, to maintain functionality (Gleitz et al., 1993; Begley et al., 1998). Cryoloading was evaluated in three steps. First, we used imaging to detect uptake of test alien substances of varying size and composition. Second, we used depolarization/extracellular Ca^2+^-dependent styryl dye uptake to confirm that SV recycling persisted in individual cryoloaded SSMs, and finally, we cryoloaded alien membrane-impermeant compounds that are known to block Ca^2+^-gated transmitter release to demonstrate utility of the method. In addition, we report a method to identify individual SSM types by their associated postsynaptic receptor types.

## Methods, results and discussion

To develop an SSM cryoloading protocol we began with methods in several previous reports that have reported retention of functional properties with SSM freeze/thaw and a single report that described SSM loading. Our main challenges were achieving maximal functional SSM retention and loading through the freeze/thaw cycle together while using minimal buffer volumes. The latter is essential for the method to be generally utile since many of the compounds that we had in mind for cryoloading, such as synthetic peptides, bacterial fusion proteins etc., are expensive or in short supply. Clumping, a centrifugation hazard also had to be avoided. While SSMs in clumps were functional, they precluded imaging of individual terminals to monitor styryl dye recycling. The resulting cryoloading method is robust; has been used reliably in over 40 experiments to date, and is practical with small quantities of test substance, as illustrated below.

### Preparation of fresh synaptosomes

**Figure 1:**
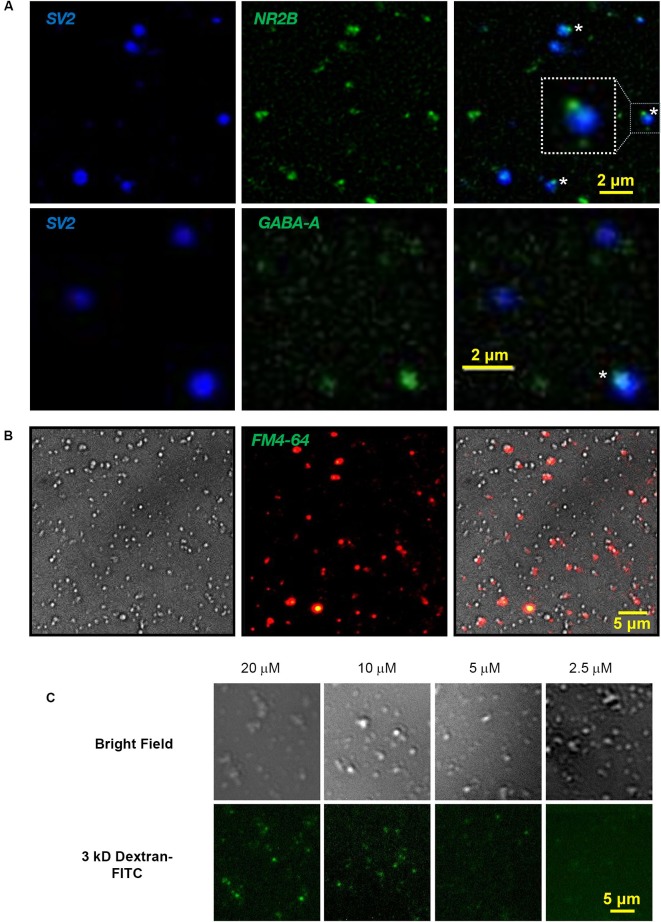
**Cryoloading introduces alien compounds into the nerve terminal.** **(A)** Fresh chick brain synaptosomes were plated on coverslips, fixed and immunostained for SV2 (DSHB, 1:1), a SV marker, and either the NMDA receptor subunit, NR2B (Abcam, ab65783; 1:200; *upper panel*) or GABA-A α1 receptor (Millipore 06-868 1:200, *lower panel*), as labeled. Staining overlays are shown on the *right*. SV2 stains SVs selectively and fills the presynaptic terminal. NMDA and GABA-A receptor antibodies identify small patches on the SSM surface (*, enlarged inset), marking the scab of postsynaptic membrane (see also Figure 2A). Light microscopy was carried out on a Zeiss Axioplan upright microscope with a 63X oil immersion, 1.45 NA objective. Image stacks used for fixed samples, as in A, were deblurred by iterative deconvolution using the Zeiss turnkey software, as described (Li et al., 2004). **(B)** Nomarski bright field *(left)*, FM4-64 uptake (2 mM Ca^2+^/40 mM K^+^; *center*) and overlay (*right*) identifying terminals with functional SV recycling in the SSM fraction. FM dye stained SSM images were background corrected using the Zeiss software function. **(C)** Chick synaptosomes cryoloaded with 3 kDa dextran-FITC with the marker at the indicated concentrations in the freezing buffer imaged by bright field (*upper panel*) and fluorescence microscopy (*lower panel*).

**Figure 2:**
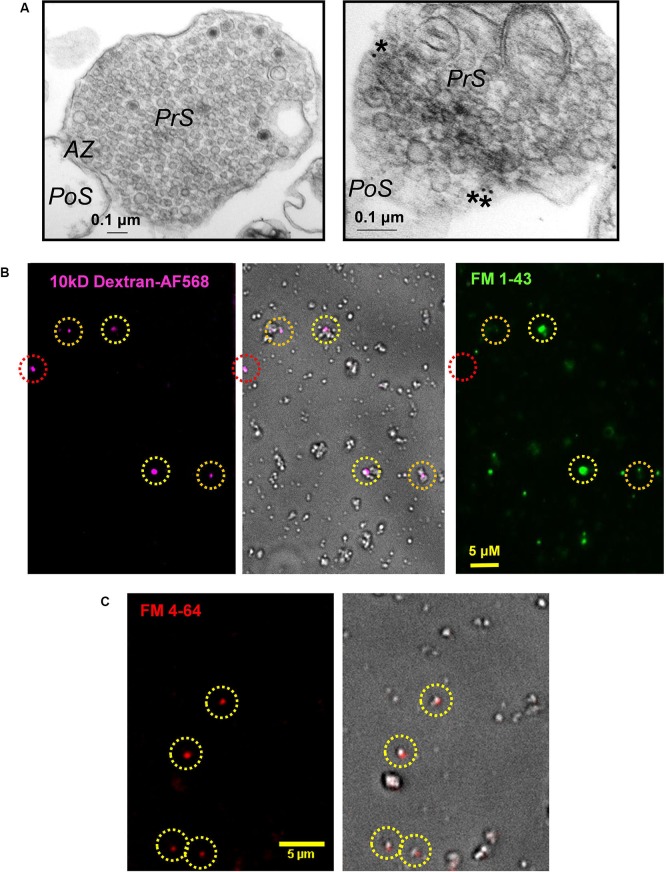
**Cryoloaded terminals are capable of SV recycling.** **(A)** *Left panel*: Electron micrographs of a control presynaptic terminal (PrS) filled with SVs and dense cytoplasm. The active zone (AZ) abuts a scab of postsynaptic membrane (PoS). *Right panel*: Electron micrograph of a colloidal gold-cryoloaded SSM. Gold particles (*) can be seen within the SSM cytoplasm. See text for methods. **(B)** Chick synaptosomes cryoloaded with 10 kDa AF568-dextran (*left*) with a bright-field overlay (center) and FM1-43 loading (*right*). Cryoloaded SSMs are indicated by dashed circles with robust (*yellow*), detectable (*orange*) or absent (*red*) FM dye staining. In control terminals the majority of cryoloaded SSMs exhibited at least detectable FM dye uptake (*see text*). **(C)** Dye uptake is retained through the cryoloading procedure. FM4-64 uptake was performed on fresh SSMs that were then subjected to the freeze-thaw cycle prior to mounting on a coverslip and imaging. The circled SSMs retained styryl dye staining (*left panel*, bright field overlay, *right panel*), indicating that recycled SVs survive the cryoloading procedure.

Fresh SSMs**** were prepared from 30 whole E15 chick brains as described (Juhaszova et al., 2000; Khanna et al., 2007; Wong et al., 2013). Briefly, the chick brains were first homogenized in homogenization buffer (HB: 0.32 M sucrose; 2 mM EDTA; 10 mM HEPES). After a slow speed spin for 15 min at 1000 x g (g-max), retaining the supernatant, followed by wash by two high-speed spins for 35 min at 250,000 x g, retaining the pellet, the cell membrane/organelle suspension was fractionated on a discontinuous sucrose gradient spun at 100,000 x g. The 0.8/1.2 M interface was collected and respun at 20,000 x g for 30 min. This SSM pellet was resuspended in 2.5 ml Sucrose, EDTA, Tris (SET) buffer (0.32 M sucrose; 1 mM EDTA; 5 mM Tris (adapted from Daniel et al., 2012)), protein concentration was determined by Bradford assay and the SSMs were diluted to 2 mg/ml (stock SSMs). SSM enrichment has been confirmed extensively by biochemical analysis (Juhaszova et al., 2000; Khanna et al., 2007; Wong et al., 2013). SSMs were plated on cover slips and were fixed and immunostained (methods: Li et al., 2004) with several presynaptic markers including SV2 (Figure 1A), CaV2.2, CSP and SNAP-25 (*not shown*). SSMs are known to comprise not only the presynaptic terminal but also a variable sized “scab” of the postsynaptic apparatus (Whittaker, 1993; see Figure 2A). We reasoned that the scab would contain residual postsynaptic receptors characteristic for the synapse type and, hence, that these could serve to identify individual SSMs. To our knowledge this has never been done previously. Thus, immunostaining for NMDA or GABA-A receptors identified SSMs with small, but intensely stained SSM-associated patches (Figure 1A) and confirmed that our SSM preparation was heterogeneous and contains both excitatory-glutamatergic and inhibitory-GABAergic terminals.

### Synaptic vesicle recycling FM1-43/4-64 styryl dye assay

We verified that the fresh SSMs were functional by testing for depolarization/Ca^2+^-dependent SV recycling using the styryl dye uptake method (Figure 1B). SSMs were plated on a coverslip in a Ca^2+^-containing Krebs-like physiological buffer (KPB: 143 mM NaCl, 4.7 mM KCl, 1.3 mM MgSO_4_, 1.2 mM CaCl_2_, 20 mM HEPES, 0.1 mM NaH_2_PO_4_, 10 mM glucose; pH 7.4). The SSMs were then exposed to the same buffer but with high K^+^ (40 mM) with FM1-43 (or FM4- 64; 1 μM) for 2 min at 30°C. SSMs were rinsed in KPB with Advasep-7 to quench residual extracellular dye fluorescence and dye uptake was imaged by fluorescent microscopy (Figure 1B).

### Sample optimization for freezing

Development of a utile cryoloading method required optimization of several aspects of the SSM sample: the quantity of stock SSMs per sample, the volume of freezing buffer, and centrifugation methods that avoid excessive SSM clumping. We settled on 100 μl of the 2 mg/ml stock for each 50 μl cryoloading sample, pelleting the stock at 13,000 RPM (16,000 x g) for 3 min at 4°C (Sorval Hereaus Fresco Tabletop Centrifuge).

### Synaptosome freeze-thaw

Each stock pellet was resuspended in 50 μl of cryoloading buffer (CB; 5% V/V DMSO in SET buffer) and maintained for 3 min at room temperature (~22°C). The target loading compounds were dissolved or dispersed in CB at a high concentration, in volumes ranging from 1 to 6 μl, and were added to the SSM suspension immediately prior to freezing. We compared two freezing methods. For “rapid-freeze” we immersed the tube in dry ice-cooled isopropanol, and for “slow-freeze” the tube was sealed in a Styrofoam container with parafilm (Sigma-Aldrich) before placing the container in a −80°C freezer. While both methods could be used, the slow-freeze method was favored because rapid-freeze resulted in a high degree of clumping of the thawed SSMs.

We also tested two methods of thawing: “rapid-thaw” with the sealed tubes in a standard cell culture incubator at 37°C for 2 min, and “slow-thaw” by incubating the tubes at 4°C (Nichols et al., 1989). SSM survival was invariably better with the rapid-thaw method. The thaw process was terminated by addition of 100 μl ice cold SET buffer to the sample. Reconstituted SSMs were then pelleted by a 2 min spin as above. SSMs that had been subjected to the freeze-thaw cycle were indistinguishable from fresh terminals by eye under the light microscope.

### Alien compounds were cryoloaded into the synaptosomes

We used inert fluorescent dextran (3 or 10 kDa; Invitrogen) as the initial cryoloading test compounds. The intensity of SSM staining varied with the concentration of the marker (Figure 1C), consistent with bulk uptake of extracellular medium. To identify the intracellular destination of cryoloaded substances, we froze SSMs in the presence of 10 nm colloidal gold (EY Laboratories). The SSMs were pelleted, fixed, stained with 2% osmium tetroxide in 0.1 M cacodylate buffer for 1 h, *en bloc* stained with 1% uranyl acetate in water for 1 h, dehydrated and then embedded, thin sectioned and mounted by standard methods. Sections were imaged on a Hitachi HT7000 transmission electron microscope operating at 75 kV. After the freeze-thaw cycle SSMs were morphologically intact with dense clusters of SVs and active zones that generally retained an attached postsynaptic fragment (Figure 2A), as described (Whittaker, 1993). Gold particles observed within SSMs were located in the cytoplasm (*N* = 3 terminals; Figure 2A, * right panel*).

### Cryoloaded synaptosomes were functional and capable of vesicle cycling

**Figure 3:**
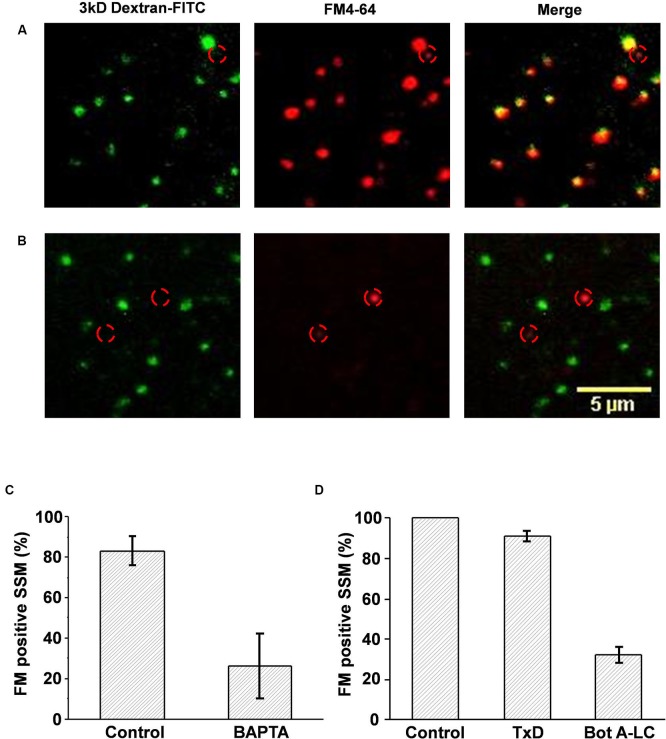
**Cryoloaded compounds are functionally active. (A, B)** Chick synaptosomes cryoloaded with plain FITC-dextran **(A)** or dextran with BAPTA **(B)** and then depolarized in the presence of FM4-64. In the absence of BAPTA all the cryoloaded (dextran positive, *left panel*) terminals also took up the styryl dye (*middle panel*), as shown in the overlay (*right panel*) demonstrating active SV recycling. As expected, one terminal that was not cryoloaded also took up dye (*location indicated by red circle in all panels*). BAPTA cryoloaded, dextran-positive synaptosomes did not take up FM4-64, demonstrating block of SV recycling. Note that non-cryoloaded neighboring terminals (*identified by red circles*) were styryl dye positive, confirming that recycling block was specific. **(C)** Histogram comparison of control, dextran-, or dextran/BAPTA-loaded SSMs that stained positive for SV recycling. **(D)** Histogram comparison of the number of SSMs that stained positive for FM4-64 after dextran-control, dextran/inactive Bot A toxoid, or dextran/active Bot A-LC cryoloading. FM uptake for each experiment was normalized to untreated controls. There was a marked reduction in the fraction of SSMs that stained for FM4-64 after Bot A-LC.

The styryl dye uptake method was used to test if cryoloaded SSMs were functional (Figures 2B, 3A). We froze SSMs in the presence of 3 kDa-Fluorescein isothiocyanate (FITC) or 10 kDa-AF568 dextran to tag the cryoloaded sub-population, using a complimentary FM dye for SV recycling assay (FM4-64 and FM1-43, respectively; Figures 2B, 3A). No dye uptake was observed without both Ca^2+^ in the extracellular medium and K^+^ depolarization. However, most (~80%, Figure 3C) of the cryoloaded SSMs exhibited K^+^/Ca^2+^-dependent styryl dye uptake, confirming that the nerve terminals were viable.

### Synaptic vesicles can be FM labeled before cryoloading

We reasoned that since styryl dyes are internalized within the SV and not the cytoplasm, FM treated SSMs should retain the dye after the freeze-thaw cycle. We confirmed that this was the case: SSMs loaded with FM4-64 (40 mM K^+^, 5 min) retained the dye after being subjected to the cryoloading procedure (Figure 2C). This observation is important. FM uptake after cryoloading requires the full SV recycling mechanism and, hence, inhibition could be due to exocytosis, endocytosis or SV transport. The pre-cryoloading FM uptake method permits a test of the effects of intracellular alien compounds that interact solely with the exocytosis arc of the SV cycle.

### Cryoloaded compounds are active in the synaptosome cytoplasm

Thus far we have demonstrated that substances can be loaded into the SSM cytoplasm and that the SSM retains its functional capacity to recycle SVs. We next tested if the cryoloaded compounds were biologically active using two membrane-impermeant compounds: BAPTA (Invitrogen), a calcium scavenger and botulinum A toxin light chain (Bot A-LC; Calbiochem), which cleaves a soluble NSF attachment protein receptor (SNARE) protein, SNAP-25, essential for SV exocytosis. Cryoloaded SSMs were identified by co-loading fluorescent dextran, as above.

#### Block of synaptic vesicle recycling by a cytoplasmic Ca^2+^ scavenger

After entering the cytoplasm via the calcium channel, Ca^2+^ ions diffuse a very short distance before binding to the calcium sensor that gates SV fusion (Stanley, 1993). BAPTA is a membrane-impermeant Ca^2+^** chelator with a binding “on” rate that is sufficiently fast and has a sufficiently high affinity to capture the ion before it can reach the sensor to block transmission (Adler et al., 1991). FM uptake assays demonstrated a marked inhibition of SV recycling in the BAPTA (30 mM) loaded terminals (26.2 ± 16.1% FM positive SSMs, *n* = 3 experiments) compared to controls (carrier alone; 83.1 ± 7.3%, *n* = 3; *p*_paired *t*-test_ < 0.05; Figures 3B, C). Thus, cryoloaded BAPTA blocked SV recycling and was active.

#### Block of synaptic vesicle recycling by an intracellular toxin

The main purpose of the cryoloading method is to introduce large peptides into the terminal. We used botulinum A light-chain (Bot A-LC) as a test. Whole Bot A toxin blocks synaptic transmission by first accessing the nerve terminal cytoplasm and then by cleaving SNAP-25 (Schiavo et al., 1995). These two functions are achieved by separate peptides: the heavy chain creates a pore for membrane penetration allowing cytoplasm access for the light chain (~50 kDa) with the enzymatic activity. Hence, the light chain is biologically inactive unless artificially introduced into the cytoplasm. We cryoloaded Bot A-LC (0.2 μM), identifying loaded terminals with co-loaded dextran 3 kDa-FITC. This was compared to two controls: dextran alone or dextran with inactivated botulinum A toxoid (TxD; 0.2 μM; Metabiologics), normalizing to the dextran-alone terminals. While there was a small reduction in the mean fraction of SSMs that were FM loaded in the TxD loaded SSMs, this failed to reach significance (91.1 ± 2.3%; *n* = 3 experiments; *p*_paired *t*-test_ > 0.05). In contrast, dye uptake was markedly reduced in the Bot A-LC loaded terminals (32.0 ± 4.0%; *p*_paired *t*-test, toxoid_ < 0.01, *p*_paired *t*-test, untreated_ < 0.01; Figure 3D). Thus, this experiment confirmed both that it is possible to cryoload large peptides and that these retain their biological activity.

Previous experimental analyses of SV recycling have used three main methods. First, injection of the compounds into presynaptic terminals that have unusually large presynaptic terminals, such as at the squid (Miledi, 1969; Llinas et al., 1981) and lamprey axon giant synapses (Martin and Ringham, 1975; Low et al., 1999), or chick (Stanley, 1989; Stanley and Goping, 1991) and rat (Stanley and Goping, 1991; Forsythe, 1994; Watanabe et al., 2010) calyceal synapses (Chen et al., 2002). While effective, these are generally experimentally challenging and laborious, prone to false negatives and because of their small size, can not be applied to the large spectrum of normal CNS bouton presynaptic terminals. Second, is to make the test compound membrane permeant, such as with the addition of an Acetoxymethyl (AM) group (Tsien, 1981). However, these are limited by the type of compound that can be carried and by possible biological activity of the tag itself, not to mention confidence that loading occurred. The third alternative has been to use genetic means to induce the neuron to express proteins and then assay the outcome using either styryl dyes or evoked postsynaptic electrical responses. This method has advantages that include application to virtually any synapse and also the deletion of existing proteins. However, these are countered by a highly involved and unpredictable preparatory effort; aberrations due to secondary changes during the expression; a requirement for the protein to be transported to the terminal, and often confidence that the intended perturbation has occurred as predicted.

The main advance of our project is the development of a very simple method to reliably introduce virtually any molecule into the cytoplasm of viable identified SSMs. The idea of loading compounds into synaptosomes by freeze-thaw was introduced by Nichols (Nichols et al., 1989; see also: Tamura et al., 2001). Those studies demonstrated calcium-sensitive transmitter efflux, detected as radioisotopes after freeze thaw. However, the reported freeze-thaw methods were not optimized nor tested for efficacy and the bulk-assay method used could not distinguish the actual source of the isotope: whether from actual cryoloaded SSMs, frozen, but unloaded SSMs, or even ruptured SSMs. While we recognize the innovation of our predecessors for the concept, we argue that by monitoring individual SSMs and tagging loaded terminals with a fluorescent marker (dextran), we present the first definitive demonstration of freeze-thaw-loading of active compounds into functional synaptosome presynaptic terminals.

It is reasonable to presume that compounds enter the terminals during cryoloading by bulk flow. Thus, at least for the small compounds, the cytoplasmic concentration should be equal to the extracellular solution concentration. However, future studies will be necessary to calibrate the degree of equilibriation in order to predict the final intracellular concentration. The method permits the introduction of peptides, proteins or markers followed by the evaluation of presynaptic function by fluorescent tagging or other methods and works equally well in other species such as rodent (*data not shown*). We also report a novel method to identify individual SSMs by their postsynaptic receptor types, making it possible (by post-recycling immunostaining) to correlate SV recycling kinetics with the specific type of SSM. We anticipate that this method will be of particular interest in the exploration of disorders that affect specific neuron types, such as dopaminergic terminals in Parkinson’s disease (Picconi et al., 2012) or cholinergic ones in Alzheimer’s (Yao et al., 2003). Thus, the cryoloading method opens up the use of the full spectrum of CNS presynaptic terminals for SV recycling analysis and can be carried out with standard fluorescence microscopy. A particular advantage of the method is that once loaded the frozen synaptosomes can be preserved almost indefinitely for future re-testing or new experiments. The method can be readily adapted to examine terminals in a specific brain region, such as cortex, cerebellum, striatum or basal ganglia, simply by selection of the starting material.

## Conflict of interest statement

The authors declare that the research was conducted in the absence of any commercial or financial relationships that could be construed as a potential conflict of interest.
